# Drug-Resistant Early-Onset Progressive Myoclonic Epilepsy Revealing Lafora Disease: A Case Report

**DOI:** 10.7759/cureus.102334

**Published:** 2026-01-26

**Authors:** Natasa Pejanovic-Skobic, Sijana Demirovic, Ana Boban Raguz, Marija Bender, Davor Batinic, Nikolina Pravdic

**Affiliations:** 1 Clinic of Neurology, University Clinical Hospital Mostar, Mostar, BIH; 2 Clinic for Children’s Diseases, University Clinical Hospital Mostar, Mostar, BIH

**Keywords:** drug-resistant epilepsy (dre), early-onset myoclonic epilepsy, lafora disease, myoclonic epilepsy, progressive myoclonus epilepsy

## Abstract

Lafora disease is a rare, autosomal recessive progressive myoclonic epilepsy characterized by drug-resistant seizures, myoclonus, and cognitive decline. We present the case of a 25-year-old woman with an unusually early onset of epilepsy at three years of age, progressive neurological deterioration, and a positive family history of progressive myoclonic epilepsy. The patient developed multiple seizure types, including generalized tonic-clonic seizures, atonic seizures, and stimulus-sensitive myoclonus, accompanied by progressive cognitive impairment. Electroencephalography (EEG) demonstrated generalized epileptiform discharges with frontocentral predominance and photosensitivity. Brain magnetic resonance imaging (MRI) revealed periventricular and parietal white matter changes with mild white matter reduction, likely related to a perinatal hypoxic-ischemic insult. Despite extensive antiseizure medication polytherapy and vagus nerve stimulation, seizures remained refractory. Although the skin biopsy was negative, the muscle biopsy showed ultrastructural changes consistent with Lafora disease. Genetic testing confirmed a pathogenic mutation in the EPM2B gene, establishing the diagnosis. This case highlights the diagnostic challenges of Lafora disease and the importance of prioritizing genetic testing in early-onset, drug-resistant epilepsy when standard diagnostic evaluations are nondiagnostic.

## Introduction

Lafora disease is a rare and severe form of progressive myoclonic epilepsy, inherited in an autosomal recessive pattern and characterized by myoclonus, multiple seizure types, and progressive cognitive decline leading to dementia [1,2]. The disease course is marked by increasing seizure frequency and refractoriness, including generalized tonic-clonic seizures, atonic seizures, atypical absence seizures, visual seizures, and focal seizures with impaired awareness [2,3].

Lafora disease is caused primarily by mutations in the EPM2A or NHLRC1 (EPM2B) genes located on chromosome 6, encoding laforin and malin, respectively [1,3-6]. Dysfunction of these proteins leads to abnormal glycogen metabolism and accumulation of insoluble polyglucosan inclusions known as Lafora bodies, the pathological hallmark of the disease [3].

Typically, Lafora disease presents in previously healthy individuals between eight and 19 years of age, with peak onset during adolescence [2,3]. Early-onset and slowly progressive forms of Lafora disease can pose a significant diagnostic challenge, as initial clinical features may overlap with more common epileptic encephalopathies or be attributed to perinatal insults. This diagnostic delay has important clinical implications, as early recognition allows timely genetic testing, appropriate counseling, and consideration of emerging disease-modifying approaches.

While Lafora disease most commonly presents during late childhood or adolescence, markedly earlier onset is rare and may significantly alter the clinical course and diagnostic trajectory. We report a unique case of Lafora disease with seizure onset at three years of age, slow disease progression, and delayed diagnosis in adulthood, underscoring the variability of clinical presentation and the challenges in early recognition.

## Case presentation

The patient was a 25-year-old woman whose disease onset occurred at the age of three, initially manifesting as frequent daily episodes of head and shoulder nodding and sudden falls associated with myoclonic jerks. Over time, seizure frequency increased to 10 to 15 episodes per day, often accompanied by loss of consciousness and urinary incontinence. Seizures were frequently triggered by emotional stress, particularly anger, or by eye closure.

As the disease progressed, generalized tonic-clonic seizures lasting three to five minutes developed, along with seizures consistent with atonic attacks characterized by backward falls and upward eye deviation. Generalized myoclonic seizures were also observed. Urinary incontinence accompanied all seizure types.

Comorbidities included hypothyroidism and gastrointestinal complaints without an identified etiology. The patient’s siblings were healthy; however, the family history was notable for progressive myoclonic epilepsy on the paternal side.

Neurological examination revealed limited verbal communication, with appropriate responses only to simple questions, indicating cognitive impairment. Psychomotor slowing was evident, although the patient remained independently ambulatory. Repeated psychological assessments confirmed mild intellectual disability. Cranial nerve function, motor strength, reflexes, and coordination were otherwise preserved.

**Figure 1:**
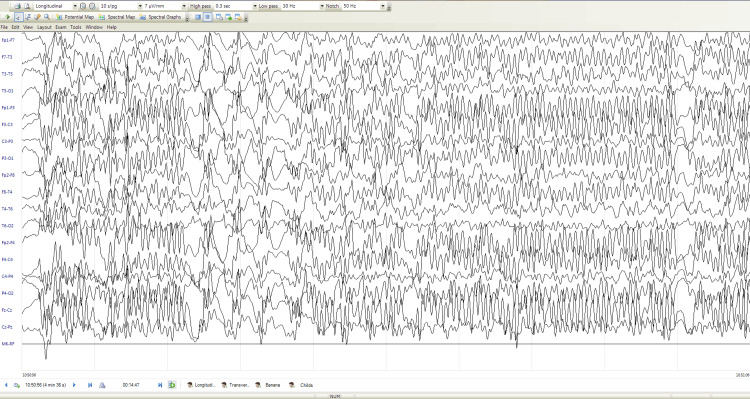
Electroencephalography (EEG) EEG recording was performed using a bipolar longitudinal montage with a sensitivity of 7 µV/mm, a high-pass filter set at 0.3 Hz, and a low-pass filter set at 30Hz. The EEG demonstrates frequent generalized epileptiform discharges with bilateral frontocentral predominance. High-voltage spikes, sharp waves, spike–slow wave complexes, and polyspikes are observed, showing a tendency toward generalization.

Electroencephalography (EEG) demonstrated diffuse paroxysmal dysrhythmic activity with spike and polyspike-wave complexes at 2-4 Hz, maximal over the frontocentral regions bilaterally, with a tendency toward generalization and marked photosensitivity (Figure 1). 

**Figure 2:**
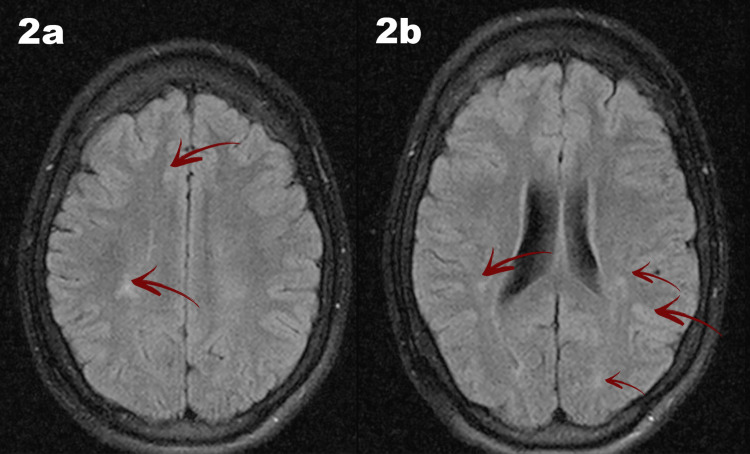
Brain magnetic resonance imaging (MRI) Brain MRI demonstrates mild asymmetry of the lateral ventricles, with the right ventricle appearing wider. Axial fluid-attenuated inversion recovery (FLAIR) images demonstrate hyperintense periventricular white matter lesions, more pronounced on the right side (Figures 2a–2b). Mild white matter volume reduction is present, consistent with gliotic changes likely related to a prior perinatal hypoxic–ischemic event. Notably, these findings occurred in the setting of an unusually early-onset and slowly progressive course, which is atypical for Lafora disease.

Brain magnetic resonance imaging (MRI) revealed mild asymmetry of the lateral ventricles, with patchy periventricular and parietal white matter hyperintensities on T2 and fluid-attenuated inversion recovery (FLAIR) sequences, accompanied by mild white matter reduction, consistent with gliotic changes likely secondary to a perinatal hypoxic-ischemic insult (Figure 2).

The patient was initially treated as having Lennox-Gastaut syndrome and underwent extensive metabolic and genetic evaluation. Metabolic studies were normal. Skin biopsy was negative for Lafora bodies. Muscle biopsy, however, revealed ultrastructural changes consistent with Lafora disease. Histopathological images from the muscle biopsy were not available for inclusion in this report. Genetic testing identified a pathogenic mutation in the EPM2B gene, confirming the diagnosis in 2015.

The patient received multiple antiepileptic medications, including valproic acid, ethosuximide, topiramate, clonazepam, lamotrigine, and later brivaracetam and phenobarbital, with persistent drug-resistant seizures (Table 1). Some of these medications had only transient or partial responses, while the majority failed to provide sustained seizure control.

**Table 1:** Antiepileptic drugs administered during the course of the disease and their corresponding dosages

Antiepileptic drug	Dosage
Valproic acid	20–40 mg/kg/day
Ethosuximide	20–30 mg/kg/day
Topiramate	3–9 mg/kg/day
Lamotrigine	1–5 mg/kg/day
Levetiracetam	20–60 mg/kg/day
Clonazepam	0.05–0.1 mg/kg/day
Clobazam	0.3–1 mg/kg/day
Vigabatrin	80–100 mg/kg/day
Gabapentin	25–40 mg/kg/day
Stiripentol	30–50 mg/kg/day
Nitrazepam	0.3–0.5 mg/kg/day
Phenytoin	4–8 mg/kg/day
Zonisamide	4–8 mg/kg/day
Diazepam	0.1–0.3 mg/kg/day
Lorazepam	0.1 mg/kg
Phenobarbital	3–5 mg/kg/day
Oxazepam	0.3–1 mg/kg/day (divided into 2–3 doses)
Acetazolamide	5–10 mg/kg/day
Brivaracetam	2–4 mg/kg/day
Corticosteroids (prednisolone)	2–4 mg/kg/day
Vagus nerve stimulation	Output current 0.25–2.5 mA

A vagus nerve stimulator was implanted in 2013, providing only mild benefit. The device became inactive in 2018 due to battery depletion and was replaced in January 2025 for reassessment of therapeutic efficacy. Corpus callosotomy was proposed as a palliative option, but was declined by the family.

Despite an early onset, the patient remains ambulatory and independent in basic self-care. Cognitive decline has progressed, with a recent evaluation showing preserved basic communication, impaired temporal orientation, reduced reading comprehension, and loss of sphincter control. Daily seizures persist, occasionally resulting in injuries.

Given the diagnostic complexity of early-onset drug-resistant epilepsy, a differential diagnostic framework was applied to guide evaluation (Table 2).

**Table 2:** Differential diagnosis considered in early-onset drug-resistant epilepsy This table summarizes the main differential diagnoses considered during the disease course, including Lennox–Gastaut syndrome, progressive myoclonic epilepsies, and metabolic or structural epileptic encephalopathies, highlighting key distinguishing clinical and electrophysiological features. Adapted from published reviews of Lafora disease and progressive myoclonic epilepsies [3]. EEG: electroencephalography

Feature	Lennox–Gastaut syndrome	Progressive myoclonic epilepsy	Metabolic/genetic epilepsies
Typical age at onset	Early childhood	Late childhood/adolescence	Variable, often in infancy
Seizure types	Multiple, tonic prominent	Myoclonic, generalized	Variable
EEG pattern	Slow spike–wave, paroxysmal fast activity	Progressive background slowing, generalized discharges	Variable
Cognitive course	Static or slowly progressive	Progressive decline	Often progressive
Genetic confirmation	Rare	Common	Common
Relevance to the present case	Initially suspected	Ultimately confirmed (Lafora disease)	Considered, then excluded

## Discussion

Lafora disease is a rare form of progressive myoclonic epilepsy characterized by treatment-resistant myoclonus, multiple seizure types, progressive neurological dysfunction, and reduced life expectancy [1-3]. Early manifestations include focal occipital seizures followed by generalized seizures, drop attacks, absence seizures, and myoclonus, while advanced stages are marked by stimulus-sensitive myoclonus, refractory epilepsy, ataxia, psychiatric symptoms, and cognitive decline [2-4].

Our patient exhibited typical features of Lafora disease, including emotion- and stimulus-triggered myoclonus, diverse seizure types, progressive cognitive impairment, and pharmacoresistance. However, disease onset at three years of age is highly atypical, as Lafora disease usually presents in late childhood or adolescence [1-3,7]. Although early-onset Lafora disease with learning difficulties has been reported [8], presentation with seizures from early childhood, as in this case, remains uncommon.

Neuroimaging in early Lafora disease is often unremarkable, with cortical or cerebellar atrophy developing later [3]. In contrast, our patient demonstrated periventricular and parietal white matter lesions and mild white matter reduction. The MRI findings were initially attributed to a possible perinatal hypoxic-ischemic insult; however, such structural changes are not characteristic of Lafora disease. In this context, the imaging abnormalities are most plausibly coincidental or represent a modifying factor of the clinical phenotype rather than a primary manifestation of the underlying genetic disorder.

In this case, the history of perinatal brain injury may have contributed to delayed consideration of Lafora disease, as early seizures and developmental impairment were initially attributed to structural brain damage. While perinatal injury may modify the clinical presentation, the progressive course, EEG evolution, and genetic findings support Lafora disease as the primary underlying etiology rather than a secondary consequence.

Brain MR spectroscopy may reveal metabolic abnormalities in Lafora disease [3,9,10] and is planned for future follow-up. The role of perinatal injury in modulating disease onset or progression remains uncertain.

Characteristic EEG findings in Lafora disease include background slowing, generalized epileptiform discharges, occipital involvement, and marked photosensitivity [3]. The EEG in our patient showed generalized high-voltage epileptiform activity with frontocentral predominance and photosensitivity, supporting the diagnosis in the appropriate clinical context.

The discrepancy between a negative skin biopsy and a positive muscle biopsy highlights known sensitivity limitations of skin sampling in Lafora disease. In the current diagnostic era, genetic testing is increasingly considered the diagnostic gold standard and may supersede invasive biopsy in suspected cases.

Management of Lafora disease is largely symptomatic. Antiseizure medications may provide transient seizure control but do not prevent neurodegeneration [2,4]. Vagus nerve stimulation has shown benefit in some patients with Lafora disease, improving seizure burden and myoclonus [11,12], with evidence suggesting increased efficacy over time [13]. Although the initial response in our patient was limited, vagus nerve stimulation therapy was reinitiated to reassess the potential long-term benefit.

Lafora disease is primarily caused by mutations in EPM2A or NHLRC1/EPM2B, with PRDM8 mutations recently linked to early-onset forms [14]. While skin biopsy may aid diagnosis in resource-limited settings, genetic testing remains the definitive diagnostic method [3,4,15]. In this case, a negative skin biopsy but positive muscle biopsy and confirmatory EPM2B mutation underscore the limitations of histopathology and the central role of molecular diagnostics.

At early stages of the disease, the diagnosis was guided by the patient’s age at onset, seizure semiology, and neurodevelopmental delay, leading initially to consideration of Lennox-Gastaut syndrome and structural epileptic encephalopathy. Lafora disease was not suspected early due to the unusually young age at onset, slow progression, and the presence of perinatal brain injury, which confounded the diagnostic process. Based on current classifications, this case is best categorized as an atypical, early-onset form of Lafora disease with a slowly progressive course extending into adulthood.

The main limitation of this report is its single-case design; however, the detailed longitudinal clinical, electrophysiological, and genetic data provide clinically relevant insights that may inform earlier diagnostic consideration and future research directions.

This case highlights the need to consider Lafora disease in young patients with drug-resistant epilepsy and cognitive decline, even in the presence of atypically early onset and slow progression. Early genetic evaluation is essential for accurate diagnosis, counseling, and emerging targeted therapeutic strategies.

Emerging therapeutic strategies for Lafora disease include gene-targeted approaches and glycogen-modulating therapies aimed at reducing Lafora body accumulation. Although these treatments remain investigational, earlier recognition of atypical presentations may enable the timely inclusion of patients in future clinical trials.

## Conclusions

Lafora disease should be considered in patients with early-onset, drug-resistant epilepsy, stimulus-sensitive myoclonus, and cognitive decline, even when disease progression is slow and initial diagnostic tests are inconclusive.

Improved awareness of atypical early-onset presentations may facilitate earlier diagnosis and inclusion of patients in future studies exploring emerging targeted and disease-modifying therapies for Lafora disease. Early genetic testing is essential for accurate diagnosis, timely counseling, and appropriate management.

In this case, despite very early disease onset and long-standing drug-resistant epilepsy, the patient has survived into adulthood, remains ambulatory, and retains partial independence in basic self-care. However, she continues to experience frequent seizures and cognitive impairment, highlighting both the phenotypic variability of Lafora disease and the limitations of current therapeutic options.
